# Assessment of the Deformability and Velocity of Healthy and Artificially Impaired Red Blood Cells in Narrow Polydimethylsiloxane (PDMS) Microchannels

**DOI:** 10.3390/mi9080384

**Published:** 2018-08-02

**Authors:** Liliana Vilas Boas, Vera Faustino, Rui Lima, João Mário Miranda, Graça Minas, Carla Sofia Veiga Fernandes, Susana Oliveira Catarino

**Affiliations:** 1 Microelectromechanical Systems Research Unit (CMEMS-UMinho), University of Minho, 4800-058 Guimarães, Portugal; liliana.sv.boas@gmail.com (L.V.B.); id5778@alunos.uminho.pt (V.F.); gminas@dei.uminho.pt (G.M.); 2 Instituto Politécnico de Bragança, ESTiG, C. Sta. Apolónia, 5300-253 Bragança, Portugal; cveiga@ipb.pt; 3 MEtRICs, DEM, University of Minho, 4800-058 Guimarães, Portugal; rl@dem.uminho.pt; 4 CEFT, University of Porto, 4000-008 Porto, Portugal; jmiranda@fe.up.pt

**Keywords:** biomicrofluidics, red blood cells, deformability, velocity

## Abstract

Malaria is one of the leading causes of death in underdeveloped regions. Thus, the development of rapid, efficient, and competitive diagnostic techniques is essential. This work reports a study of the deformability and velocity assessment of healthy and artificially impaired red blood cells (RBCs), with the purpose of potentially mimicking malaria effects, in narrow polydimethylsiloxane microchannels. To obtain impaired RBCs, their properties were modified by adding, to the RBCs, different concentrations of glucose, glutaraldehyde, or diamide, in order to increase the cells’ rigidity. The effects of the RBCs’ artificial stiffening were evaluated by combining image analysis techniques with microchannels with a contraction width of 8 µm, making it possible to measure the cells’ deformability and velocity of both healthy and modified RBCs. The results showed that healthy RBCs naturally deform when they cross the contractions and rapidly recover their original shape. In contrast, for the modified samples with high concentration of chemicals, the same did not occur. Additionally, for all the tested modification methods, the results have shown a decrease in the RBCs’ deformability and velocity as the cells’ rigidity increases, when compared to the behavior of healthy RBCs samples. These results show the ability of the image analysis tools combined with microchannel contractions to obtain crucial information on the pathological blood phenomena in microcirculation. Particularly, it was possible to measure the deformability of the RBCs and their velocity, resulting in a velocity/deformability relation in the microchannel. This correlation shows great potential to relate the RBCs’ behavior with the various stages of malaria, helping to establish the development of new diagnostic systems towards point-of-care devices.

## 1. Introduction

Malaria is a parasitic disease with more than half the world population at risk and around 500 thousand deaths per year, with 80% of infections occurring in children under 5 years old [1]. This disease is mainly widespread in underdeveloped regions, with lack of proper infrastructure and living conditions, worsening the chances of infection for the population. The control, effective treatment, and elimination of malaria require an early and accurate diagnosis. Currently, the malaria diagnosis is based on blood smear microscopy or rapid diagnostic tests (RDTs) [2,3], which have limitations in the detection limit (only detect above 50 parasites/µL of blood). Additionally, microscopy has limitations in the required time to perform the assays and in the need for specialized technicians and/or laboratories, compromising the reduction of global incidence. To fulfill these needs, innovative diagnosis based on molecular assays have been developed, with detection limits below 2 parasites/µL, particularly using loop-mediated isothermal amplification [4] or more advanced portable devices such as QuantuMDx/Q-POC [5]. However, these techniques require disposable reagents, technicians, more than 30 min to get the test results, and imply aseptic conditions (hard to maintain in endemic regions). Therefore, there is a huge need for fast, reagent-free, and low-cost malaria diagnostic systems, without requiring special training and independent of the genetic variability of the parasite, and overall the final ideal device should comprise all these concerns.

The malaria parasite lifecycle passes from the mosquito vector to the human host by entering the liver cells where it matures, to further being released into the blood stream, invading the red blood cells (RBCs). At this stage, the infected RBCs (iRBCs) suffer biochemical, optical, and morphological changes [6,7], making these cells more rigid and thicker, resulting in a decrease of the cells velocity (when the cells are infected with *Plasmodium falciparum* parasite) [8]. Hemodynamic studies help to obtain information regarding the evolution of the disease. Particularly, the RBCs’ deformability and the RBCs’ velocity when crossing a geometric contraction can work as relevant markers for malaria diagnostics applications, since they are directly related to the changes that the parasite causes throughout the evolution of the disease [9]. The literature reports different methods for assessment of the RBCs’ deformability, including filtration [10], ektacytometry [11,12], optical tweezers [13,14], micropipette aspiration [15], and microfluidic geometrical constrictions [16,17,18,19,20,21,22]. Some numerical and experimental studies in the literature already report the relation between RBCs’ deformability and hemodynamics [23,24], or between deformability and the individual RBCs’ velocities in specific geometrical conditions [9,25,26,27,28]. This work will be focused on a microfluidic system to measure the RBCs’ deformability and velocity, as well as to establish a relation between these properties when the cells cross geometric microcontractions, with the expectation to, in the future, compare this correlation with the real malaria effects in RBCs. The microfluidic systems are a potential alternative to the current diagnostic methods, since they are able to mimic the hemodynamic phenomena that happens in blood vessels and have advantages in terms of sample preparation and analysis (low volume of samples, easy handling, low-cost, and fast processing), eliminating the need for specialized personnel [22]. Additionally, microfluidic devices enhance the possibility of creating a fully automated and portable diagnostic device for malaria, when assembled in a microfluidic platform that includes microfluidic handling, control and readout electronics, and data acquisition.

In order to develop and evaluate those microfluidic methods for the deformability and velocity assessment, it is essential to synthetically impair the RBCs for mimicking malaria behavior, for testing the method’s efficiency and reproducibility, without the constant need for parasites or infected samples, improving laboratorial safety, when testing, and decreasing the costs. For that purpose, glutaraldehyde, diamide, and glucose will be used for increasing the rigidity of the RBCs and, their effect in narrow constrictions will be compared [29,30,31,32]. When exposed to these chemicals, the RBCs will be rigidified and their dynamic behavior in narrow constrictions, relative to deformability and velocity, will be compared to healthy RBCs. The evaluation of the RBCs’ velocity and deformability will be performed in a set of microchannels with abrupt constrictions, followed by abrupt expansions [25]. This approach takes advantage of the potential of these sudden geometrical contractions to deform the cells due to shear and extensional flows. The cells’ behavior will be captured by a setup comprising a high-speed camera and a microscope, and the obtained images will be processed in two software tools (ImageJ and PIVLab) for determining both the RBCs’ deformability and the RBCs’ velocities, as well as determining the relationship between those properties.

## 2. Materials and Methods 

This section presents the materials and samples used to perform the experimental assays, as well as the description of the microchannel fabrication method, experimental setup, and image processing techniques. In brief, RBC samples with low concentration (low hematocrit) will be exposed to glutaraldehyde, diamide, or glucose and will be tested in polydimethylsiloxane (PDMS) microchannels that comprise 8 µm widths abrupt contractions. The ability of the RBCs to flow through the microchannels contractions will be assessed.

### 2.1. Microchannels Fabrication

**Figure 1:**
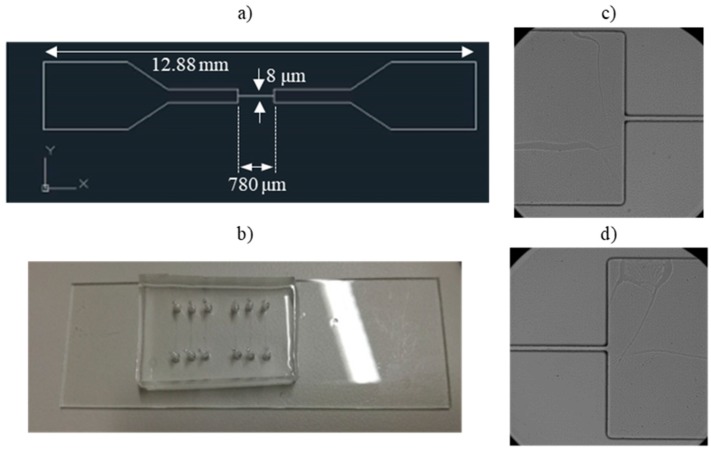
(**a**) 2D masks for microchannel fabrication. The narrow contractions in the central region of the microchannels have 8 μm width; (**b**) polydimethylsiloxane (PDMS) microchannels with a 12.8 mm total length; (**c**) Detail of the entrance of the 8 μm width contraction of the PDMS microchannel; (**d**) Detail of the outlet of the 8 μm width contraction of the PDMS microchannel. Magnification: 40×.

A polydimethylsiloxane (PDMS) microfluidic device was microfabricated by soft lithography techniques, using SU-8 molds (SU-8 purchased from Microchem Corporation, Westborough, MA, USA) [33,34]. PDMS (Sylgard 184 Silicone Elastomer kit obtained from Dow Corning, Midland, MI, USA) was chosen due to its transparency that is required for microscope visualization, easy fabrication, and low-cost for prototypes. The PDMS microchannels have a 25 µm height in order to reduce the flow volume and the number of RBCs within the microchannels, also making it easier to observe the RBCs. Each microchannel is composed by a linear transition zone followed by an abrupt contraction (at a 90° angle) with 8 µm width and 780 µm length (seen in Figure 1), designed to force the RBCs to deform and gain velocity when crossing it. The width of the contractions mimics capillary vessels with the same average dimensions of the RBCs (around 8 µm).

### 2.2. Samples

For the in vitro assays, samples containing human RBCs (hematocrit = 0.5%) in Dextran40 (Dx40) were used. Human RBCs have a biconcave shape and typical diameters in the 6–8 µm range, being highly deformable.

The healthy human whole blood samples were taken from a female volunteer and provided by Instituto Politécnico de Bragança (Bragança, Portugal). All procedures for the blood collection, transport, and in vitro experiments were carried out in compliance with the EU directives 2004/23/CE, 2006/17/CE, and 2006/86/CE and approved by the Unidade Local de Saúde do Nordeste (Bragança, Portugal). In order to evaluate the RBCs’ deformability and velocity in the microchannels, the RBCs were separated from the other blood constituents through centrifugation (15 min, 2000 rpm, at room temperature). After that, RBCs were re-suspended and washed twice in a physiological solution (PSS) (from B. Braun Medical, Melsungen, Germany) with a NaCl concentration of 0.9%. The Dx40 solution, where the RBCs were suspended, was used as a plasma-volume expander to prevent RBC sedimentation and maintain the ideal osmotic physiological conditions for the RBCs. This solution was synthetically produced by mixing 68 μL of CaCl_2_ with 201 μL of KC, 7.35 mL of NaCl, and 5 g of Dx40 (for 1 M solution) (all reagents purchased from Sigma-Aldrich, St. Louis, MO, USA). The 0.5% hematocrit, representing a 0.5% volume of RBCs in 5 mL of Dx40, was considered in order to assure that the RBCs are isolated when crossing the microchannel contraction. Although the 0.5% hematocrit is significantly lower than the physiological one, it was decided to study diluted samples, to improve the visualizations and measurements of each individual RBC and, as a result, to avoid effects such as interactions and aggregation of RBCs. Preliminary tests performed with hematocrit values ranging from 0.5% up to 2% have shown that as the concentration of RBCs was increased, it was difficult to individually follow the RBCs and, consequently, to measure the RBCs’ velocity and deformation index. Hence, the current study was performed with a hematocrit of 0.5%.

The RBC samples were then modified with glucose (COPAN Diagnostics Inc., Murrieta, CA, USA), glutaraldehyde (Sigma-Aldrich Corporation, St. Louis, MO, USA), and diamide (Sigma-Aldrich Corporation, St. Louis, MO, USA) solutions, in order to rigidify the cells at different levels. These chemicals were selected since they are commonly used to perform deformability studies, are accessible, and have simple preparation protocols, as well as they allow one to rigidify the cells at different levels, according to the added concentration. To modify the RBCs with glucose, four different concentrations of glucose were considered: 2%, 5%, 10%, and 20% (*v*/*v*). First, glucose (powder) was diluted in a phosphate buffered saline solution (PBS: pH 7.4). Then, the RBCs (already separated from the other blood constituents and suspended in Dx40) were incubated for 20 min, at room temperature, at each of the referred glucose concentrations. The cells were then washed in PSS to remove the excess of glucose from the samples and re-suspended in Dx40. To modify the cells with glutaraldehyde, at 0.00625%, 0.0125%, 0.025%, and 0.08% glutaraldehyde concentrations (*v*/*v*), the RBCs (already separated from the other blood constituents and suspended in Dx40) were incubated for 10 min at each of the referred concentrations, washed in PSS, re-suspended in Dx40, and used right away. The RBCs were also modified with diamide, at 0.00625%, 0.0125%, 0.025%, 0.08%, 0.32%, and 1% diamide concentrations (*v*/*v*), using the same protocol: Incubation for 10 min at each of the referred concentrations, washing in PSS, and re-suspension in Dx40.

### 2.3. Experimental Setup

The cells’ deformability and velocity assays were performed with an experimental setup comprising the microfluidic device placed on the stage of an inverted microscope (IX71; Olympus Corporation, Tokyo, Japan). A flow rate of 5 μL/min was controlled using a syringe pump system (KD Scientific Inc., Holliston, MA, USA). For selecting the ideal flow rate, preliminarily studies were performed for four different flow rates (0.1, 1, 3, and 5 µL/min) and no significant differences were observed in the cells’ deformability. Additionally, it was observed that the syringe pump system presented more stability for the highest tested flow rate, i.e., the 5 μL/min. The images of the RBCs were captured using a high-speed camera (Fastcam SA3, Photron, Motion Engineering Company, Westfield, IN, USA) at a 2000 frames/s rate and exported to a computer to be analyzed. Each assay was repeated 3 times.

### 2.4. Image Processing and Analysis Techniques

**Figure 2:**
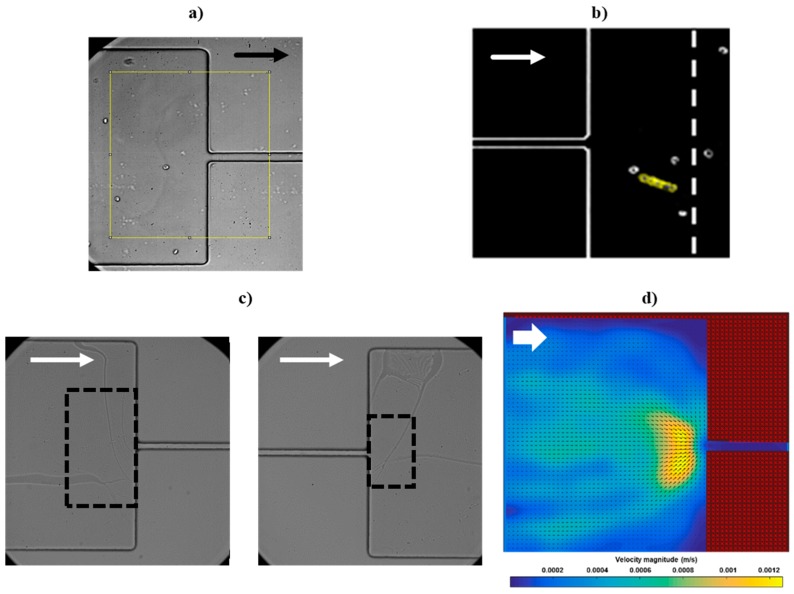
(**a**) Example of a cut-off of a transfer zone (308 µm × 332 µm) in the entrance of the microchannel contraction, using the crop function of ImageJ; (**b**) Example of a tracked red blood cell (RBC) at the outlet of the microchannel contraction, using ImageJ, where the dashed line represents a region where the RBCs expand after the outlet (relaxation area); (**c**) Definition of the areas (inside the dashed lines) for measuring the RBCs’ deformability and velocity at the entrance (left—121 µm × 237 µm region) and at the outlet (right—86 µm × 142 µm region) of the microchannel contraction (Magnification: 40×); (**d**) Example of the velocity distribution, obtained with PIVLab, of healthy RBCs (non-modified) at the entrance of the microchannel contraction (the arrows indicate the flow direction in each frame). Note that, due to limitations of the available equipment (frame rate acquisition), it was not possible to acquire frames with RBCs moving at high velocity in the interior of the microchannel contraction and, as a result, no cells were registered in that region, explaining the 0 velocity in the image.

The images exported from the high-speed camera to the computer were analyzed using two software tools: ImageJ [35] and PIVLab [36,37]. For each assay, a sequence of 10,000 frames was considered. ImageJ was used to perform the pre-treatment of the acquired frames, in order to remove the noise and image artifacts, as well as convert them into binary images. Initially, the image sequence was imported and the crop function was executed to define the region of interest (ROI) as a rectangle with 308 µm × 332 µm dimension (Figure 2a). Then, by using the Z-Project function, the selected frames were stacked to determine an average of the frames. This averaged frame was subtracted from all the frames under analysis, eliminating all static objects, which resulted in frames comprising only the visible RBCs, without any additional information. Finally, by using a threshold function, the images were converted into binary images. The ImageJ software was also used to measure the cells size in order to calculate the RBCs’ deformation index (DI). Using the ROI Manager and the Measure functions, it was possible to follow both the healthy and the impaired RBCs (example in Figure 2b) and calculate their DI along the microchannel, using the expression: DI = (*X* − *Y*)/(*X* + *Y*), where *X* and *Y* represent the largest (*X*) and the smallest (*Y*) axis of the ellipse correspondent to the RBC under analysis. Typically, the RBCs’ DI varies between 0 and 0.8, where 0 represents non-deformed cells and 0.8 represents cells at maximum elongation. For each assay, a group of RBCs was followed at the entrance and at the outlet of the contraction to measure their DI and determine an averaged value. Figure 2c presents the area at the entrance and at the outlet of the microchannel contraction (the areas inside the dashed lines in Figure 2c), where the deformability of the RBCs is measured. These areas were chosen after performing preliminary observations of the RBC flows. For the entrance of the contraction, a 121 µm × 237 µm region of interest was selected, since it is in this area that the RBCs experience the highest extensional flow and consequently start to deform to enter the narrowing. For the outlet, in the region immediately after exiting the contraction, the RBCs are at maximum deformation, and outside that region, the cells start to recover their original shape. Then, for assuring a standard area at the outlet for all assays, an 86 µm × 142 µm region of interest was selected. It should be noted that the evaluation area at the outlet of the contraction is significantly smaller than at the entrance. This difference is explained by the authors’ intention, in future devices and prototypes, of integrating micro-sensors in the outlet of the contraction (occupying the smallest area possible) and, therefore, in this work it was expected to obtain relevant data from a small area of evaluation in the outlet.

In order to determine the average of the velocity values of the RBCs at the entrance and at the outlet of each contraction, the sequence of frames was analyzed using the PIVLab image analysis toolbox, integrated in MATLAB. First, the pre-treated images were imported into the software and calibrated (relatively to their dimensions and time between frames), and a ROI mask was applied to remove the areas where there are no RBCs. Following, the motion of the particles between the frames was analyzed and the instantaneous velocities were calculated by the variation of the distance traveled by the RBCs between each time step. Then, the average velocity vectors (*U_x_* and *U_y_*) were calculated in the *x* and *y* directions and the velocity field of each sample (*U_xy_*) was determined based on the equation: |*U_xy_*| = sqrt (*U_x_*^2^ + *U_y_*^2^). Finally, a filter was applied to smooth the images and remove the high frequencies, which could indicate spikes of velocity without physical significance. Figure 2d presents an example of the velocity field distribution at the entrance of the contraction. It is possible to observe that the velocity of the RBCs is significantly higher in the zone of the narrowing entrance, reasoning that the abrupt transition causes an increase of the velocity of the RBCs. Note that, due to limitations of the available equipment, it was not possible to acquire frames with RBCs moving at high velocity in the interior of the microchannel contraction. As a result, almost no cells were registered in that region, explaining the 0 velocity in the interior of the contraction in the PIVLab image (Figure 2d), this way the results section will approach and compare the DI and velocity of the RBCs at the entrance and outlet of the contractions, neglecting the study of cells inside the contraction regions. Note that both RBC deformability and velocity are measured in the same area (as defined in Figure 2c, left and right) in order to establish a relation between the RBCs’ deformability and their velocity. After obtaining the velocity distribution immediately before the entrance and after the outlet of the contraction, a criterion for determining the RBCs’ velocity was defined (as presented in the Results Section 3): From the region of interest (Figure 2c, left and right), where the velocities are higher, the 100 pixels with highest velocity (obtained in PIVLab) were selected and those velocities were averaged, neglecting the surrounding areas with lowest velocities.

Additional details on the ImageJ and PIVLab procedures for the determination of RBC deformability and velocity can be found in [25].

## 3. Results and Discussion

**Figure 3:**
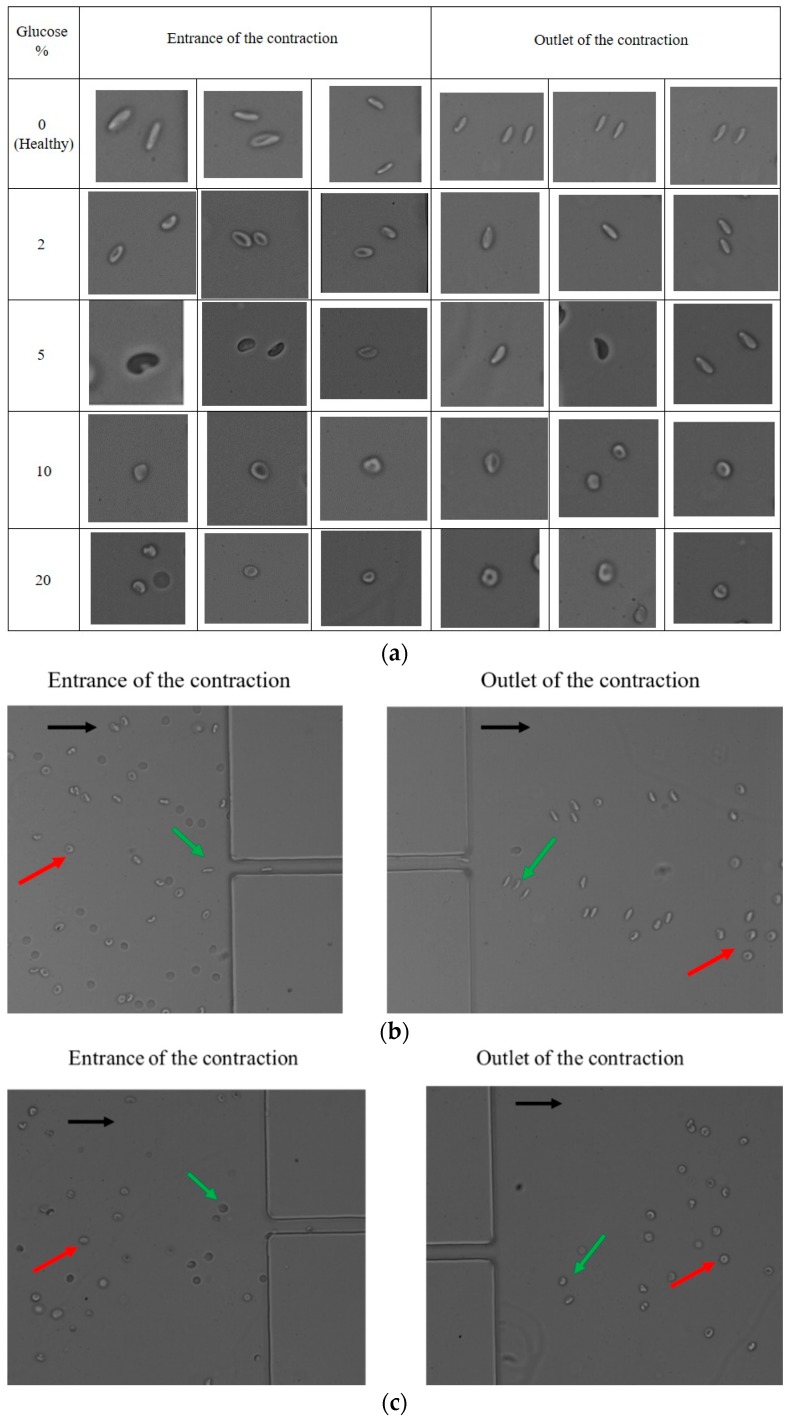
(**a**) Examples of healthy RBCs and RBCs modified with different glucose percentages at the entrance and at the outlet of the microchannel contraction, extracted from three assays; (**b**) Healthy RBCs (red arrow, left) deforming at the entrance of the contraction (green arrow, left), leaving the contraction still deformed (green arrow, right), and recovering their original shape following the outlet on an expansion area (red arrow, right); (**c**) 10% glucose-modified RBCs (red arrow, left) with almost no deformation at the entrance of the contraction (green arrow, left) and leaving the contraction (green arrow, right), recovering their original shape on an expansion area (red arrow, right). The black arrows indicate the flow direction.

This section presents the deformability and velocity results (obtained as in Section 2.4) of the comparison between healthy and chemically modified RBCs with glucose, glutaraldehyde, and diamide. All the presented results are an average of three assays. For each assay, a sequence of 10,000 frames was considered and around 10 RBCs were followed to measure their DI. Figure 3 shows examples of RBCs from different assays at the entrance and at the outlet of the contraction in the PDMS microchannel, for different percentages of glucose, considering a 5 μL/min flow rate, and for a healthy RBC sample (for control—0% glucose). From Figure 3a, it is possible to detect a difference between RBC deformability as the glucose percentage increases, i.e., the RBCs change from a deformed/stretched shape to a non-deformed shape, as the cells have more difficulties to deform and tend to keep their original shape.

These results suggest that the glucose concentration affects the RBCs’ deformability, in agreement with several past studies regarding the influence of glucose over RBC deformability [31,32]. The increase of glucose (hyperglycemia) in RBCs causes damage in the RBCs’ membranes and increases the blood viscosity, also increasing the cells’ aggregation, which leads to a significant decrease on the RBCs’ DI. When the RBCs were modified with glutaraldehyde or diamide, the results were similar to the ones observed for glucose (shown in Figure 3), and, therefore, only the glucose images are presented. Following the outlet of the microchannel contraction, the RBCs start to recover their shape, again decreasing their deformation index, as shown in Figure 3b,c, for an assay with healthy RBCs and one assay with 10% glucose-modified RBCs.

**Figure 4:**
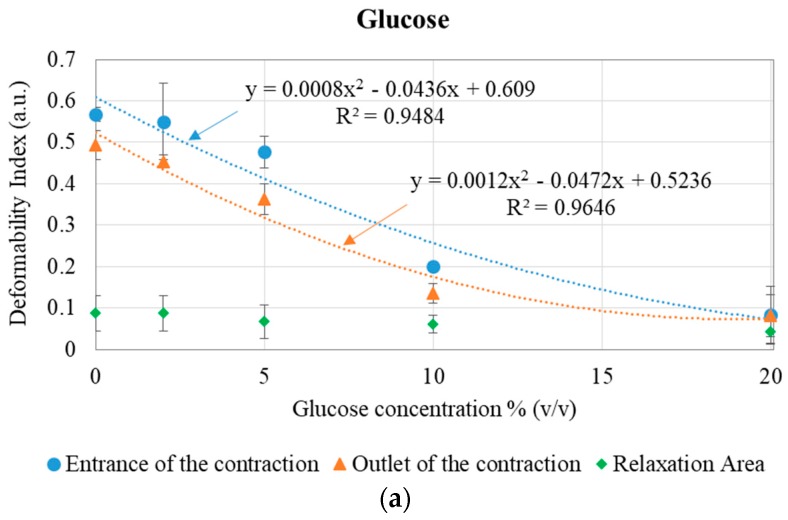

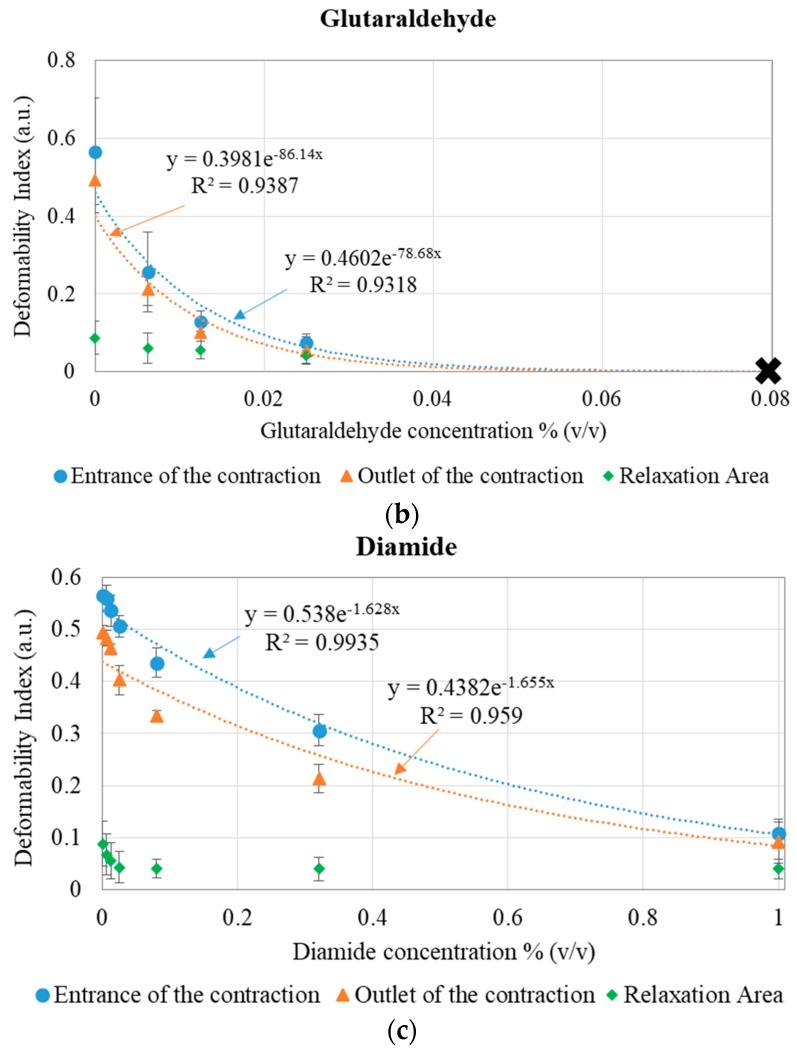
Deformation index (DI) and error bars for healthy and (**a**) glucose-, (**b**) glutaraldehyde-, and (**c**) diamide-modified RBCs, at the entrance (blue series), at the outlet (orange series) and at the relaxation area (green series) of the microchannel contraction and trend lines. In (b), the *X* represents the clogging of the microchannel, with no deformability or velocity data. Each point of the plots is the average of 30 RBCs (three assays for each condition and 10 RBCs followed in each assay).

Figure 4 presents the DI for healthy and modified RBCs (with glucose, glutaraldehyde, and diamide), at the entrance and at the outlet of the microchannel 8 µm contraction, as well as at the relaxation area (see Figure 2b), for the 5 μL/min flow rate. 

The results show that, as the percentage of glucose, glutaraldehyde, or diamide increases, the cells tend to become more rigid, decreasing their DI [29]. While the healthy cells deformed at the entrance of the contraction to pass throughout the contraction and then recovered their initial shape after reaching the microchannel expansion area, the modified RBCs did not deform and some aggregation of the cells was observed, increasing the difficulty to cross the contraction. At the outlet of the contraction, where the deformability was measured, the RBCs tend to start to recover their original shape, which is verified in Figure 4: The RBCs at the outlet have lower DI than at the entrance of the contraction. As the rigidity of the cells increases, the difference between the DI at entrance and at the outlet of the contraction decreases. Since the evaluation regions at entrance and at the outlet have a different total area (as defined in Figure 2c), it may also help to explain the hysteresis in the results between entrance and outlet (the entrance evaluation area is larger than the outlet evaluation area). 

Table 1 presents the differences between the averaged RBC deformability at the entrance of the contraction and at the relaxation area, for all the tested conditions. This allows us to observe the cells’ maximum deformability, passing from their deformed shape entering the contraction, until their recovered shape after relaxation. The results show that, as the rigidity of the cells increases, the difference in the deformability between the entrance and the relaxation area (ΔDI) decreases, and this behavior is similar for the three chemicals tested: Glucose, glutaraldehyde, and diamide.

**Figure 5:**
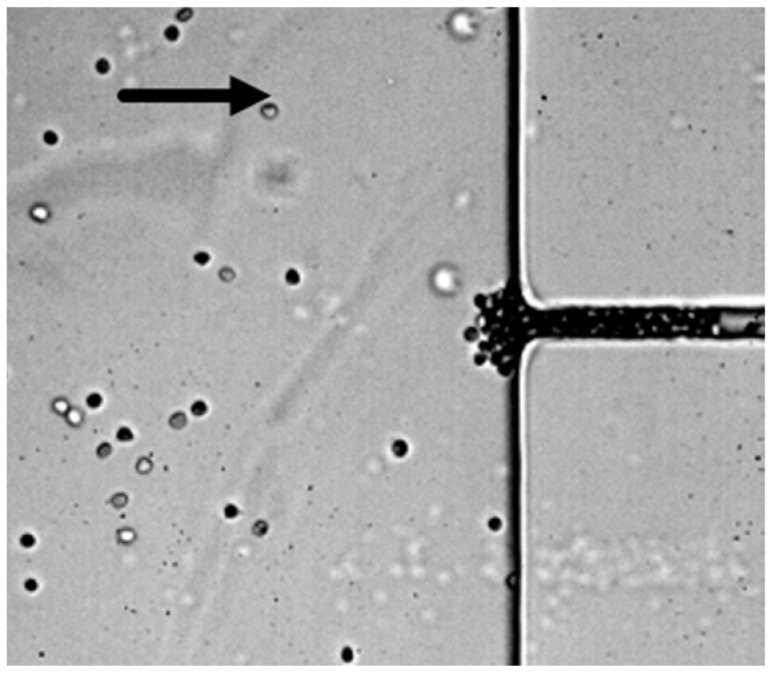
Detail of clogging at the entrance of the 8 µm contraction when the RBCs were modified with a 0.08% (*v*/*v*) concentration of glutaraldehyde.

It was also observed that, for 0.08% (*v*/*v*) glutaraldehyde-modified RBCs, the rigidified cells clogged the entrance of the contraction and no deformability or velocity data could be extracted (this is represented by *X* in Figure 4b). Therefore, for a high concentration of glutaraldehyde, it was unable to measure the transiting velocity of the cells. Figure 5 presents an example of clogging at the entrance of the contraction, when the RBCs were modified with a 0.08% (*v*/*v*) concentration of glutaraldehyde.

Elevated blood glucose in the RBCs alters RBC membrane proteins through glycosylation and oxidation. Glutaraldehyde penetrates into cell membranes and non-specifically cross-links the cytosol, the cytoskeletal, and the transmembrane proteins, acting on all components of the cell and increasing the effective viscosity of the cytoplasm and lipid membrane. Diamide is a spectrin-specific cross-linker, oxidizing thiol groups while forming disulfide bonds within the structural region [30]. The obtained results indicate that viscous effects in the cytoplasm and/or lipid membrane are a dominant factor when dictating dynamic responses of RBCs in pressure-driven flows, explaining the higher effect of the glutaraldehyde in damaging the RBCs and the microchannel clogging, when compared to diamide and glucose [30].

**Figure 6:**
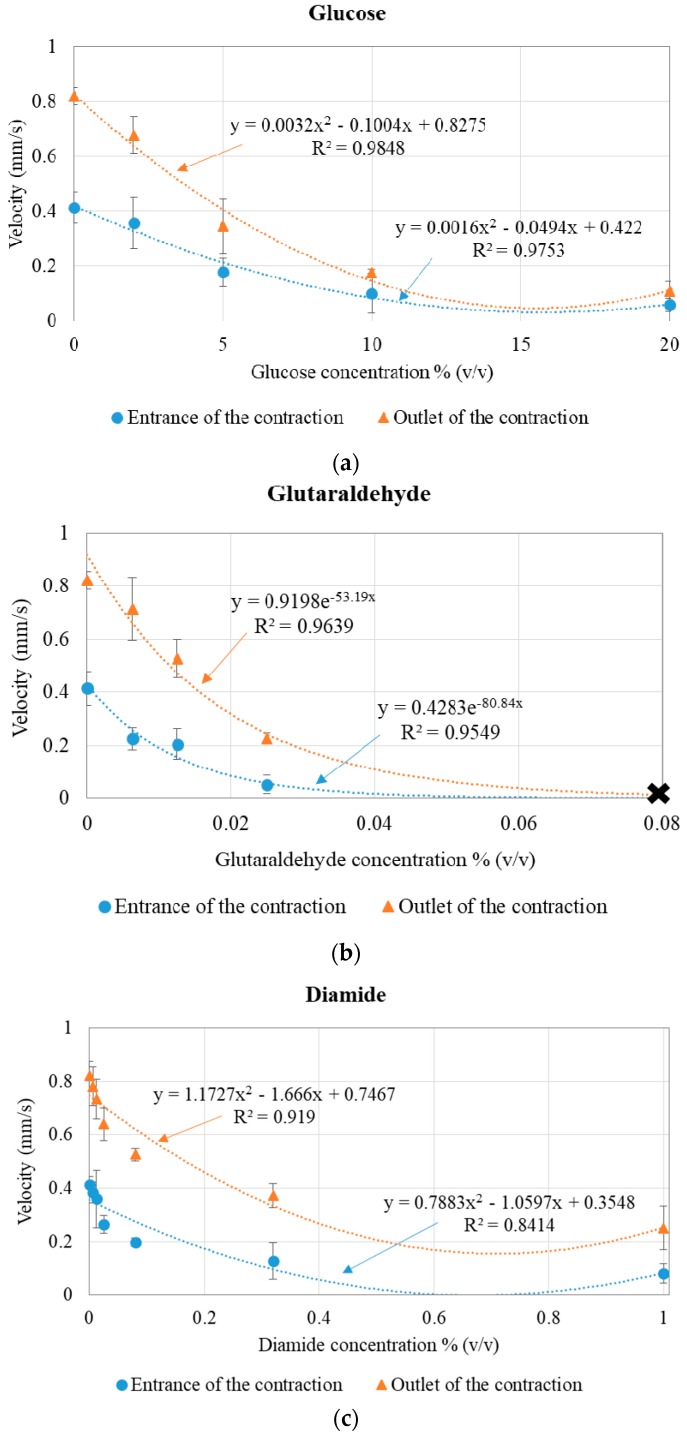
Velocity (mm/s) and error bars for healthy and (**a**) glucose-, (**b**) glutaraldehyde-, and (**c**) diamide-modified RBCs, at the entrance (blue series) and at the outlet (orange series) of the microchannel contraction and trend lines. In (b), the *X* represents the clogging of the microchannel, with no deformability or velocity data.

Figure 6 presents the average cell velocity for healthy and modified RBCs (with glucose, glutaraldehyde, and diamide), at the entrance and at the outlet of the microchannel 8 µm contraction, for the 5 μL/min flow rate.

Overall, the results agree with those of deformability. When the average velocity at the high velocity regions was evaluated, it was found that the impaired RBCs (by adding glucose, glutaraldehyde, or diamide) presented lower velocities than healthy RBCs, indicating that the increase of the RBCs’ rigidity causes the non-deformed cells to follow streamlines that on average have lower velocity, while the stretched and healthy RBCs follow streamlines that on average have higher velocity. Supplementary material videos show how the cells gain velocity when entering the contraction, explaining the higher velocity immediately at the outlet of the contraction (when compared to the velocity at the entrance), before starting to relax and recover their original shape. Similarly to the deformability results, for 0.08% (*v*/*v*) glutaraldehyde-modified RBCs, the rigidified cells clogged at the entrance of the contraction and, as a result, no velocity data could be extracted (this is represented by *X* in Figure 6b).

**Figure 7:**
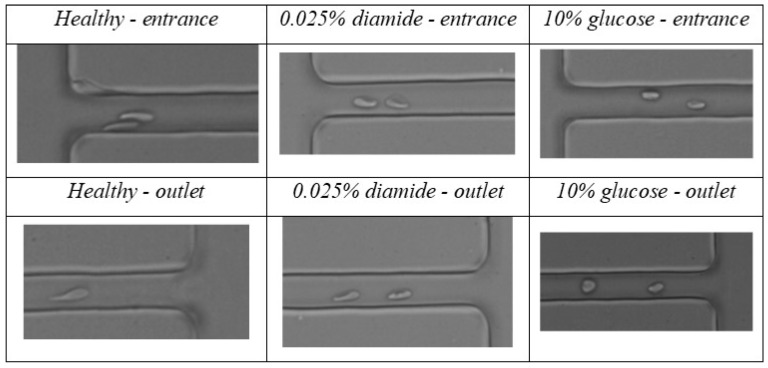
Examples of healthy RBCs, RBCs modified with 0.025% diamide, and RBCs modified with 10% glucose inside the 8 µm width microchannel contraction, at different areas (entrance and outlet of the contraction).

Additionally, it would be interesting to quantitatively study the relation between deformation index and velocity inside the microchannel contraction, besides the data presented at entrance and outlet (Figure 4 and Figure 6). However, due to technical limitations of the high speed acquisition system, it was not possible to acquire an enough number of RBCs with high quality contrast to perform the measurements of RBC deformability and velocity with the software tools referred in Section 2. Despite that limitation, some RBCs could still be observed within the contraction. Examples of RBCs (healthy, with 0.025% diamide, and with 10% glucose) flowing within the contraction are shown in Figure 7.

A qualitative analysis of the presented results shows that healthy RBCs cross the microchannel contraction in a more deformed shape than the 0.025% diamide and 10% glucose samples, and the 10% glucose samples are less deformable than the 0.025% diamide ones, which corroborates the quantitative results (before the entrance and after the outlet) presented in Figure 6. Supplementary material presents videos of healthy and modified RBCs flowing at the entrance and at the outlet of the contraction, allowing a better observation of the RBCs’ behavior at the different regions of the microchannel contraction.

**Figure 8:**
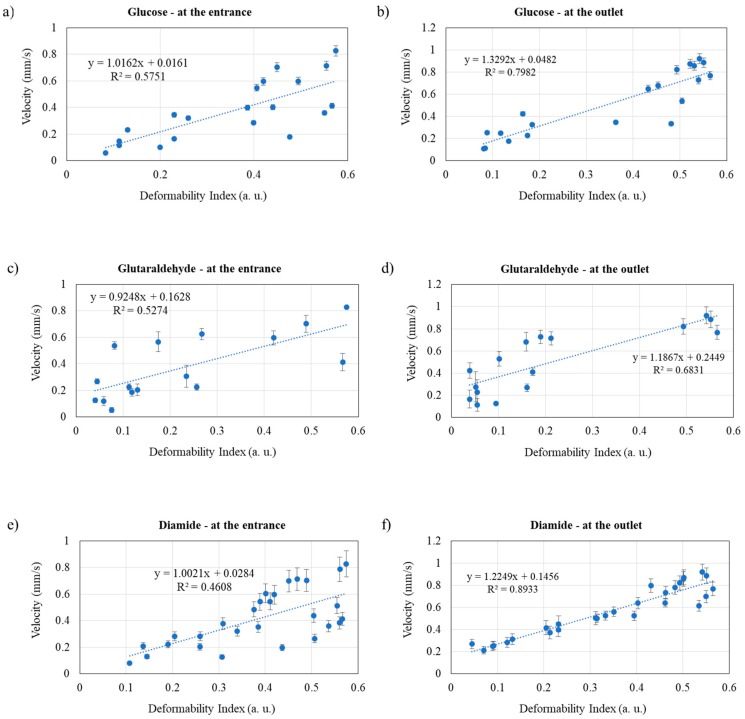
Velocity (mm/s) vs. deformability (a.u.) curve, measured at the entrance and at the outlet of the 8 µm contraction, for the RBC samples modified with (**a**) at entrance; glucose, (**b**) at outlet; glucose (**c**) at entrance; glutaraldehyde; (**d**) at outlet; glutaraldehyde, (**e**) at entrance; diamide, and (**f**) at outlet; diamide.

Since one of the main objectives of this work was to establish a relation between the RBCs’ deformability and their velocity, Figure 8 presents the velocity vs DI calibration curves for glucose-, glutaraldehyde-, and diamide-modified RBCs at the entrance and at the outlet of the microchannel contraction. This figure purpose is to show the dispersion that occurs between the cells. Therefore, instead of presenting all RBCs averaged together (as in Figure 4 and Figure 6), it is intended to evaluate how each small group of cells fits the deformability vs velocity curve, in order to understand their individualized behavior. Therefore, from the performed assays, the RBCs were gathered in groups of three cells measured under the same conditions and their average was calculated (each blue dot of the plots). Consequently, each plot of Figure 8 gathers data from a high number of RBCs (three RBCs × number of dots in each plot, leading to a range of RBCs between 3 × 16 = 48 in Figure 8d and 3 × 28 = 84 in Figure 8e), measured in the areas defined in Figure 2.

Based on the results, it is observed that, overall and as expected, for all synthetically modified RBCs, an increase of cell deformation index leads to an increase of the cells’ velocity, both at the entrance and at the outlet of a microchannel contraction. When comparing the velocity with the deformability correlation at entrance and at outlet, it is clear, for all the tested methods, that the results at the outlet present a better fitting to the linear tendency curve than at the entrance (based on the *R*^2^ values). Therefore, in the future, when advancing for a diagnostic tool, the analysis must be performed at the outlet of the contraction (the place to integrate a sensor), where the RBCs’ behavior is more reliable. Additionally, our results indicate that diamide is the most interesting approach for mimicking the malaria effects on RBCs with the intention of exploring sensor applications, as the velocity vs DI results show a better fitting to the linear tendency curve (*R*^2^ = 0.89) and, consequently, it is easier to control the velocity vs deformability curve. These results are a promising step to help the development of integrated sensors in microfluidic devices that allow the design of an autonomous malaria detection system of high sensitivity, precise, low-cost, portable, and with low energy consumption.

## 4. Future Perspectives

Future works will include an increase in cell quantity in order to define the average property of the entire cell population with higher accuracy, since the physical properties of individual RBCs within the same RBC population can vary significantly [38]. Additionally, since the ultimate goal is to develop a clinical tool, more blood samples from different donors will be assessed to increase the RBCs’ variability and to include more independent data. It is also planned to improve our high speed video microsystem, allowing the capture of good enough quality images to quantitatively measure both velocity and deformability of the RBCs flowing across the microchannel contraction. This improvement will allow to develop an improved association between RBC deformability and transiting velocity through the narrow constrictions. Finally, after obtaining an improved correlation with synthetically modified samples, it is intended to test real parasite-affected RBC samples [39] to measure their deformability and velocity, compare disease and artificially impaired RBCs, establish target values, and fully validate the proposed approach. This improved correlation will be used to relate the RBCs’ behavior according to the various stages of malaria and to develop integrated sensors in microfluidic devices for RBC velocity measurements.

## 5. Conclusions

This work has investigated the deformability and the velocity of healthy and chemically modified RBCs, attempting to mimic the effect of malaria in RBCs and to establish a relation between RBCs’ velocity and deformability. The glucose, glutaraldehyde, and diamide effect in the RBCs was compared using PDMS microchannels with 8 µm narrow contractions that forced the RBCs to undergo deformation when they passed through them. It was concluded that, by adding glucose, glutaraldehyde, or diamide, the RBC membrane tends to become stiffer, decreasing the cell’s deformability and, consequently, decreasing the cell shape recovery capacity. Additionally, when the RBCs’ rigidity increased, the RBCs’ velocity decreased. 

When the relation between deformability and velocity was evaluated, it was concluded that, for all synthetically modified RBCs, an increase of the cells’ deformation index led to an increase of the cells’ velocity. It was also verified that diamide was the most interesting approach to impair the cells and mimic the malaria effects on RBCs, as the velocity vs deformation index results have showed the best fitting to the linear tendency curve and, consequently, it would be easier to control the deformability and velocity of the cells based on this method. 

Despite still being a challenge, this work will be a valuable contribution to help establishing the development of simple, reagent-free, inexpensive, and accurate new malaria diagnostic systems towards point-of-care devices.

## Figures and Tables

**Table 1:** Difference of the deformation index (ΔDI) between red blood cell (RBC) deformability at the entrance of the contraction (Figure 2c, left) and at the relaxation area (Figure 2b) for all the tested conditions, obtained from the data presented in Figure 4.

Sample	Concentration (%)	ΔDI
Healthy RBCs	0	0.479
RBCs + Glucose	2	0.463
	5	0.410
	10	0.139
	20	0.041
RBCs + Glutaraldehyde	0.00625	0.196
	0.0125	0.074
	0.025	0.034
	0.08	*X*
RBCs + Diamide	0.00625	0.493
	0.0125	0.482
	0.025	0.464
	0.08	0.396
	0.32	0.267
	1	0.068

## Supplementary Materials

The following are available online at http://www.mdpi.com/2072-666X/9/8/384/s1, Video S1: 10% glucose RBCs at the entrance of the contraction, Video S2: 10% glucose RBCs at the outlet of the contraction, Video S3: Healthy RBCs at the entrance of the contraction, Video S4: Healthy RBCs at the outlet of the contraction.
